# Supplementary material for: Cancer patient survival can be parametrized to improve trial precision and reveal time-dependent therapeutic effects
Source: Nat Commun. 2022 Feb 15;13:873. doi: 10.1038/s41467-022-28410-9 (PMC8847344; doi:10.1038/s41467-022-28410-9)
Supplement: Supplementary file 1 — Supplementary information [file 41467_2022_28410_MOESM1_ESM.pdf]

**SUPPLEMENTARY INFORMATION (FIGURES)**

# Cancer patient survival can be parametrized to improve trial precision and reveal time-dependent therapeutic effects

Deborah Plana[1,2], Geoffrey Fell[3], Brian M. Alexander[3,4], Adam C. Palmer[5*], Peter K. Sorger[1*]

[1]Laboratory of Systems Pharmacology and the Department of Systems Biology, Harvard Medical School, Boston, Massachusetts, USA.

[2]Harvard-MIT Division of Health Sciences and Technology, Harvard Medical School and MIT, Cambridge, Massachusetts, USA.

[3]Dana-Farber Cancer Institute, Boston, Massachusetts, USA.

[4]Foundation Medicine Inc., Cambridge, Massachusetts, USA.

[5]Department of Pharmacology, Computational Medicine Program, Lineberger Comprehensive Cancer Center, University of North Carolina at Chapel Hill, Chapel Hill, North Carolina, USA.

[*]These authors contributed equally. To whom correspondence should be addressed:

palmer@unc.edu; peter_sorger@hms.harvard.edu (cc: sorger_admin@hms.harvard.edu)

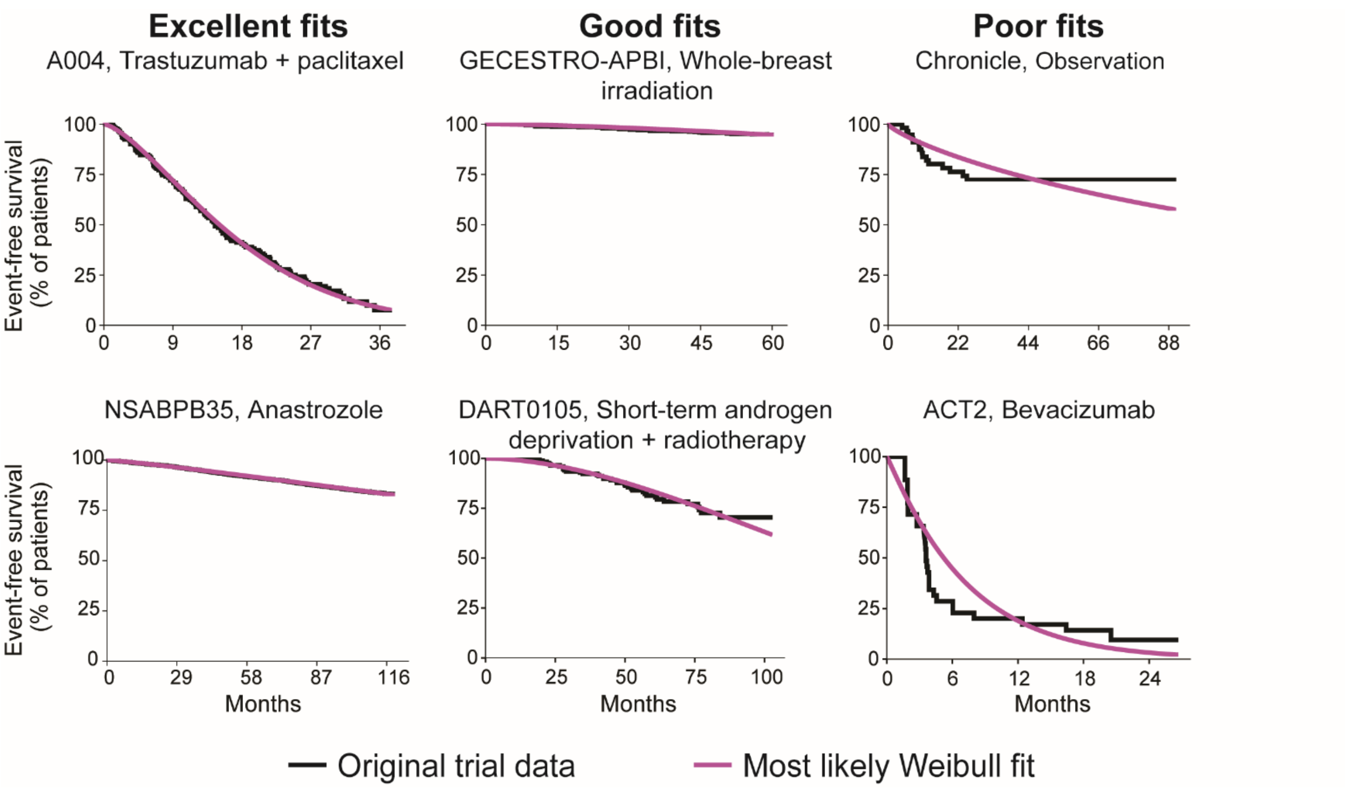


**Supplementary Fig. 1. Representative fits of Weibull distributions to event-free survival data.** Weibull fits to data for plots of the Kaplan-Meier estimator falling in the top 25$^{th}$ percentile quality of all fits (excellent fits; NCT00294996, NCT00053898), between the first and third quartile (good fits; NCT00402519, NCT02175212), and in the lower quartile (poor fits; NCT00427713, NCT01229813). Data derived from trials reporting event-free survival data (301 survival curves from 146 figures), primarily progression-free survival.

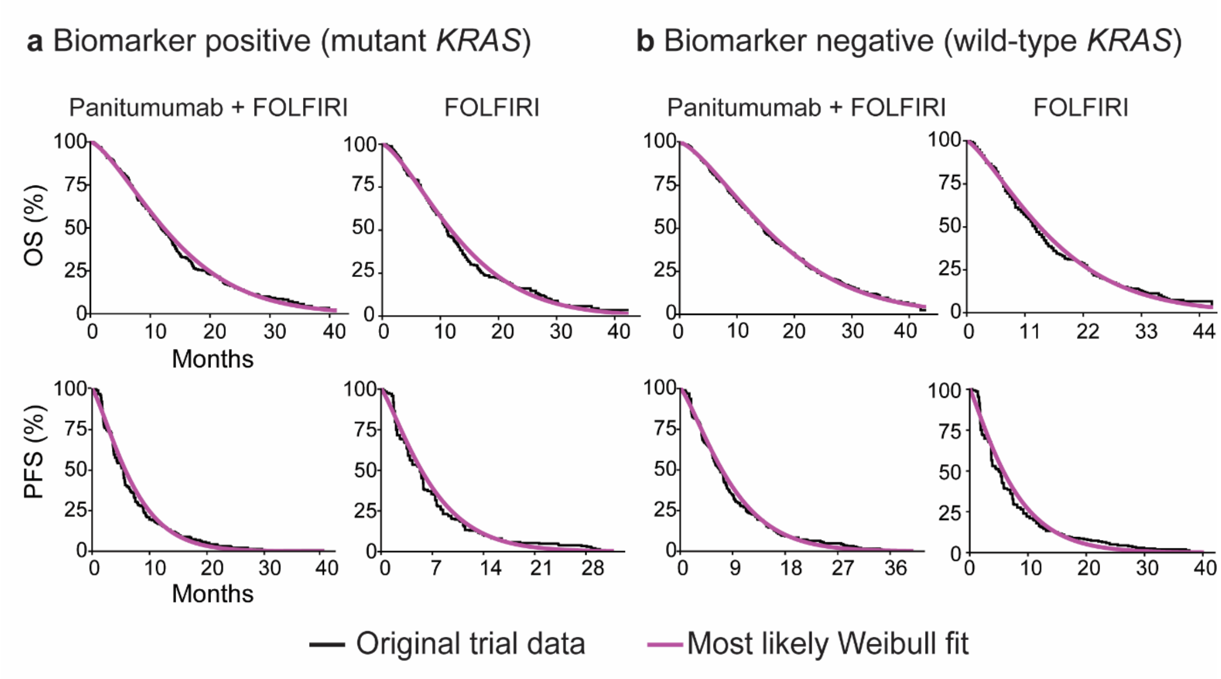


**Supplementary Fig. 2. Fit of Weibull models to overall survival (OS) and progression-free survival (PFS) data from biomarker-stratified trials.** Data obtained from the 20050181 trial (NCT number not reported in the original publication).

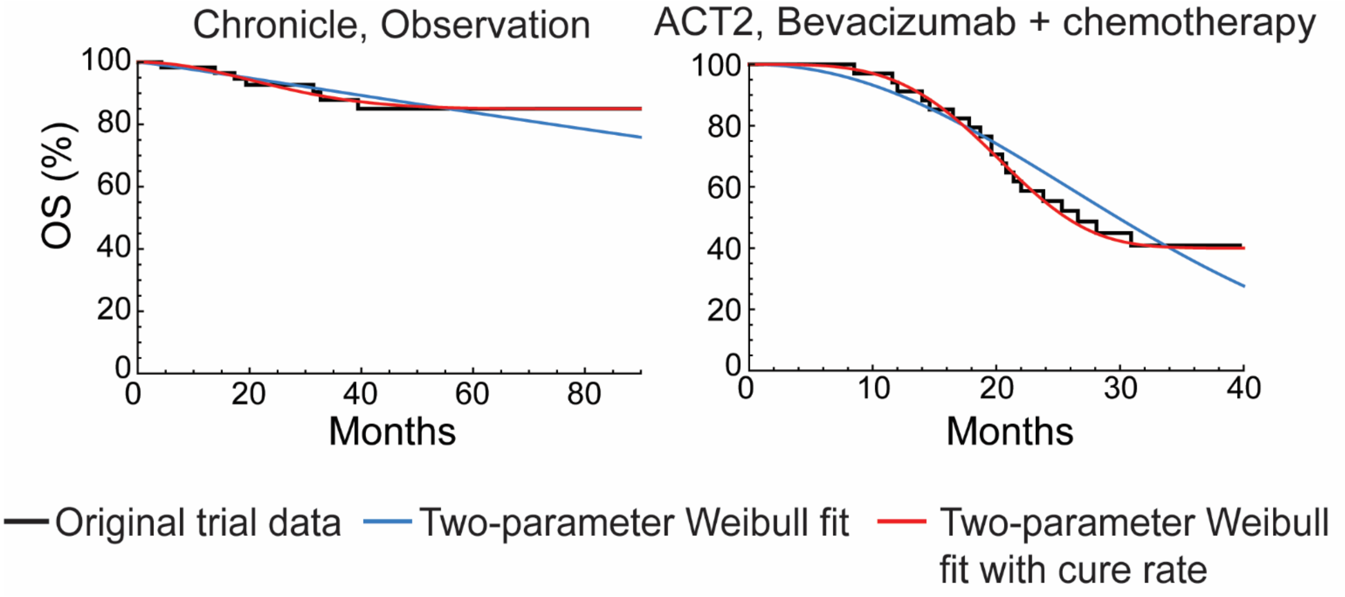


**Supplementary Fig. 3. Improvements in fit of Weibull distributions to overall survival (OS) data using a cure rate.** Two-parameter fits (NCT00427713, NCT01229813) and two-parameter fits with an additional cure rate parameter.

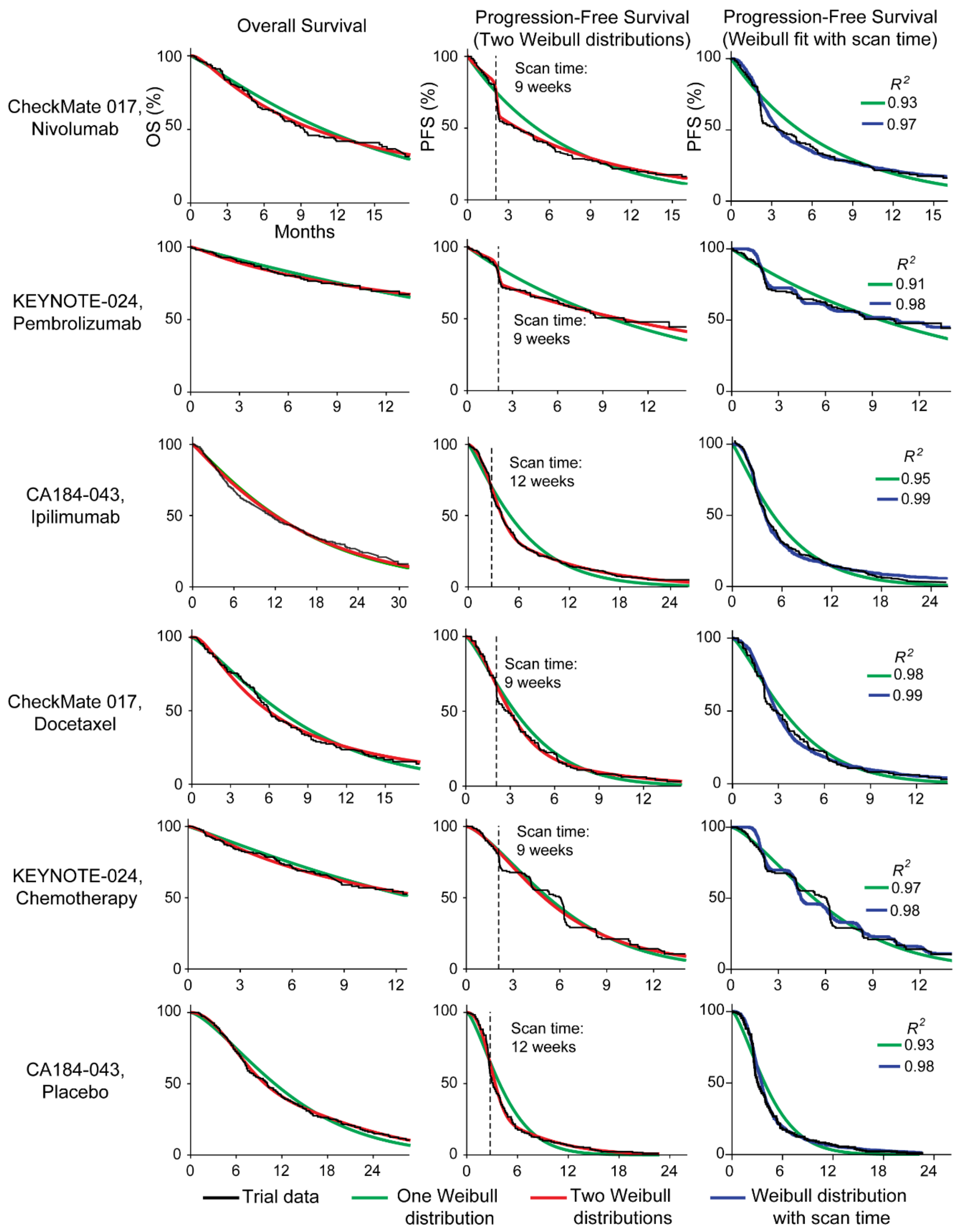


**Supplementary Fig. 4. Additional fits to overall survival and progression-free survival data for trials of immune checkpoint inhibitors.** PFS and OS distributions for three trials of immune checkpoint inhibitors with one and two-distribution fits using Weibull functions, as well as simulations that account for the periodicity of radiological scans to detect progression (NCT01642004, NCT02142738, NCT00861614).

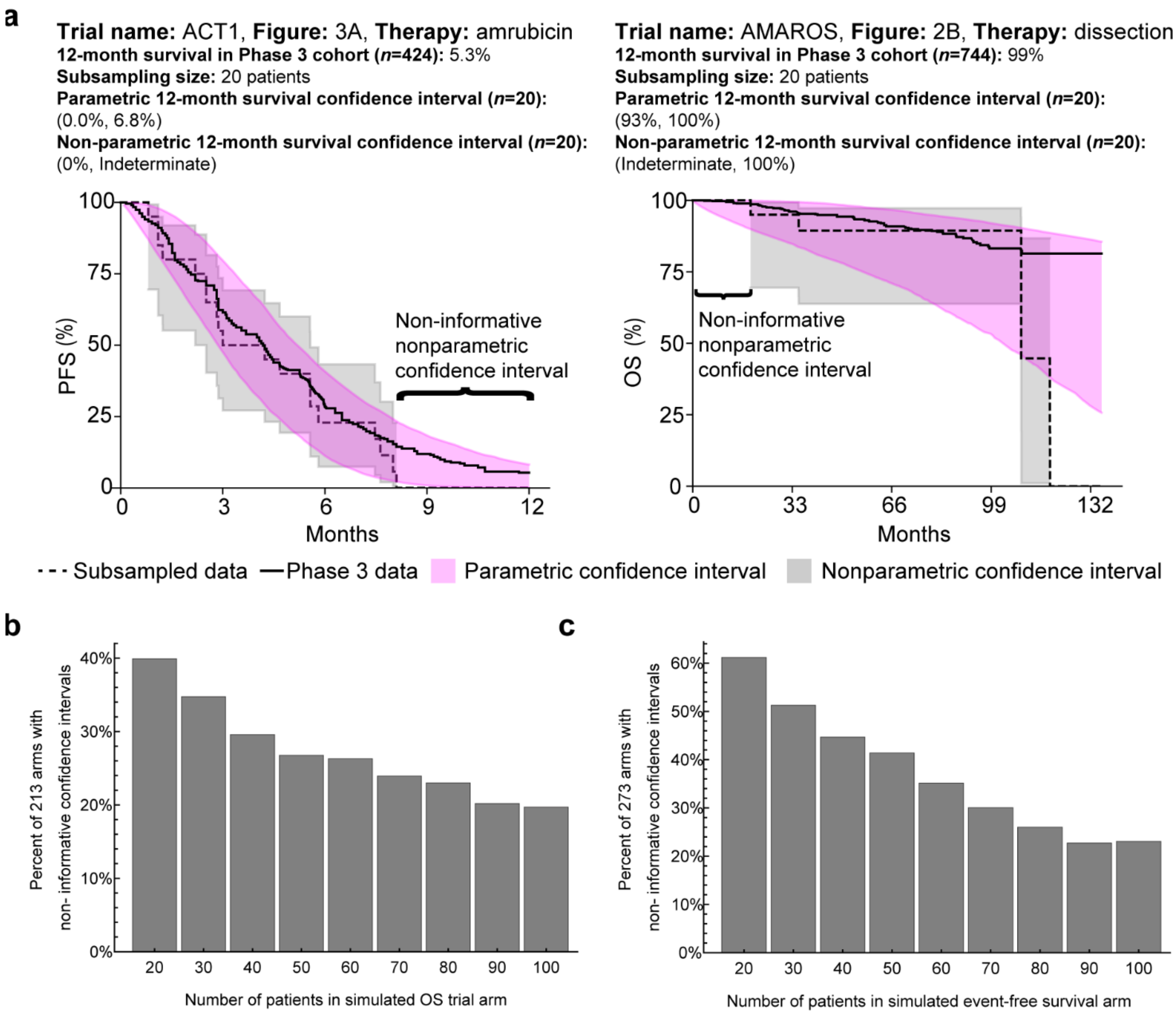


**Supplementary Fig. 5. Non-informative nonparametric confidence intervals generated from subsampled patient data. a** Example nonparametric non-informative confidence intervals for progression-free survival (PFS) and overall survival (OS). **b** Rate of non-informative, nonparametric confidence intervals generated from subsampled patient events across sample sizes for OS and **c** event-free survival. Ten simulations were performed per trial arm and sample size; a trial arm was scored as producing a non-informative confidence interval if one of ten simulations produced an “indeterminate” 12-month survival bound.

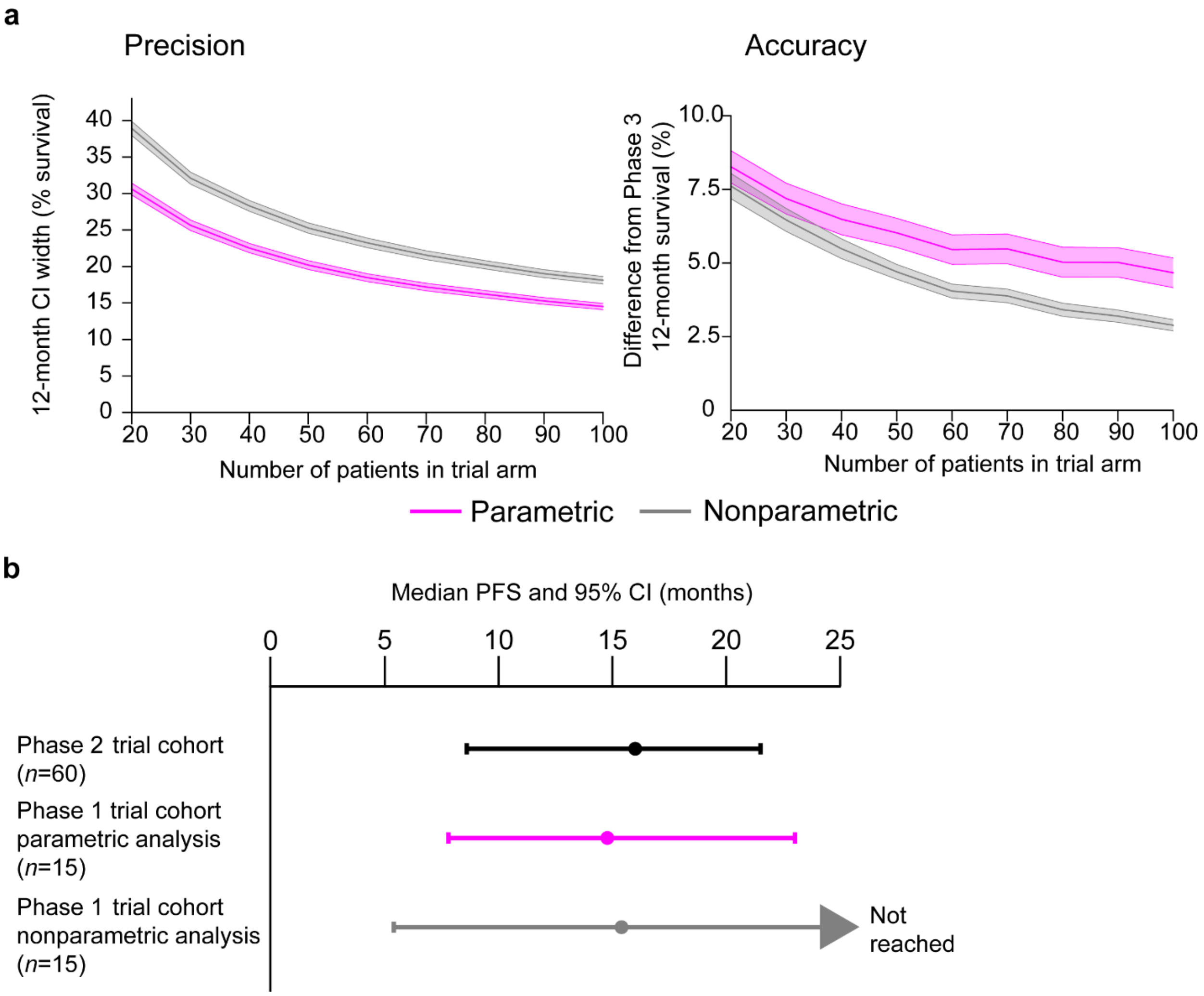


**Supplementary Fig. 6. Impact of parametric fitting on the precision and accuracy of event-free survival estimates. a** Comparison of nonparametric and parametric (Weibull distribution) methods to compute 12-month event-free survival confidence intervals (CIs) for trials with small cohorts (20 to 100 patients) produced by randomly subsampling patient events from 99 event-free survival trial arms. Note that nonparametric estimates did not return an informative CI for 64% of event-free survival curves (out of 273 trial arms with at least 100 patients and one event taking place before 12 months), while Weibull fitting made it possible to calculate 12-month CIs for every survival curve in every simulation. Precision is defined as the width of the CI in percent survival. Accuracy is defined as the absolute difference between the 12-month survival estimated from small cohorts and the value computed from all patients in a given Phase 3 trial arm. Lines denote mean values and shaded regions are 95% CIs. **b** Median progression-free survival (PFS) CIs calculated with parametric and nonparametric methods on Phase 1 clinical trial data and the corresponding Phase 2 study results (MK-3475-022/KEYNOTE-022; NCT02130466).

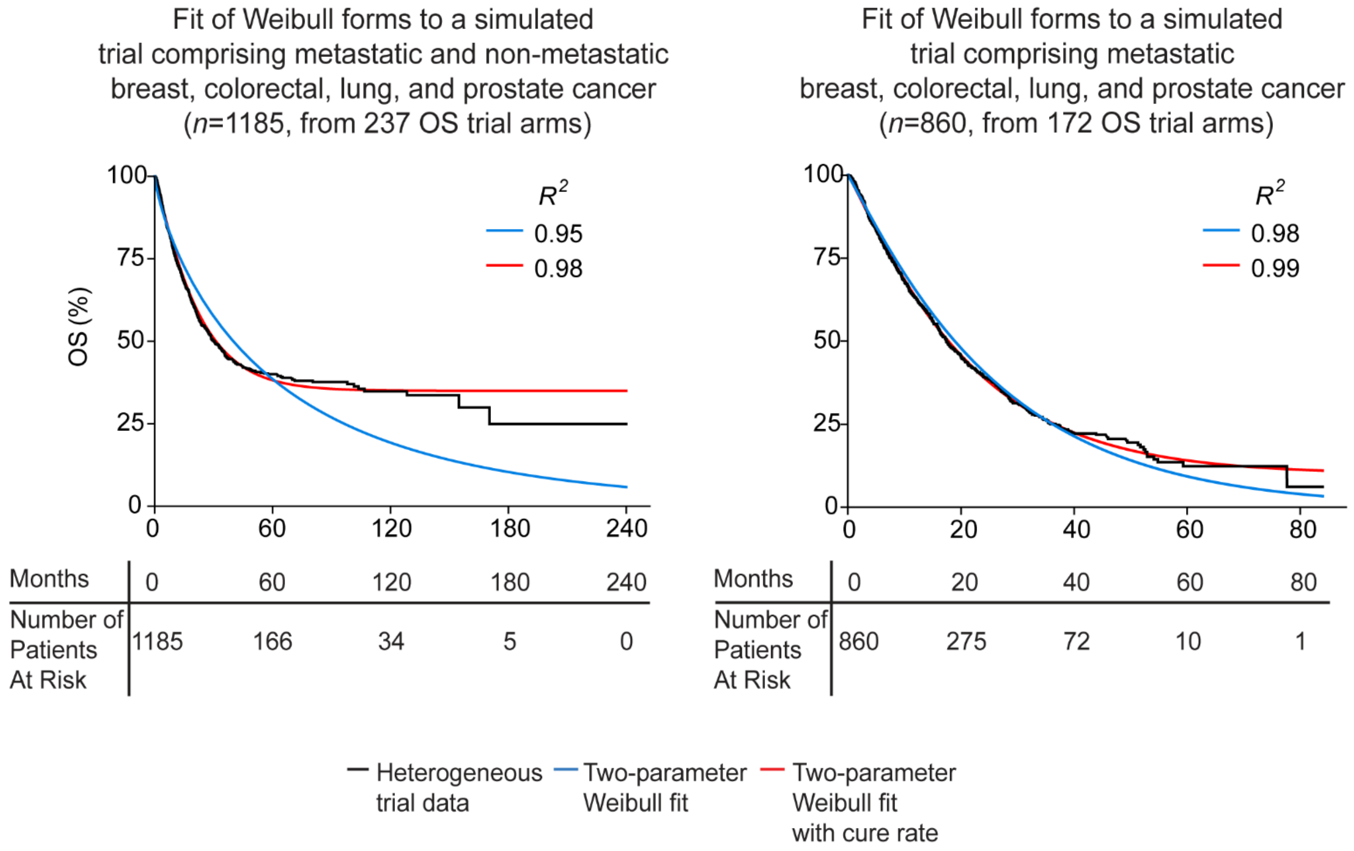


**Supplementary Fig. 7. Performance of Weibull fitting on trial arms subsampled from heterogeneous cancer trials.** Two-parameter (blue) and two-parameter with cure-rate (red) Weibull fits for simulated trial arms. The synthetic cohorts were generated by subsampling from all overall survival (OS) curves (237 arms, *n*=1185) and from OS curves enrolling patients with metastatic cancers (172 arms, *n*=860). The quality of the Weibull cure-rate fit was calculated by 1) computing the moving average of times of all deaths in the simulated trial arm, 2) scaling the observed survival probabilities at those times using the cure-rate parameter ((survival probability- cure rate)/(1-cure rate)), 3) transforming all scaled survival probabilities and corresponding times using a Weibull plot, and 4) computing an $R^2$ value as described in the Methods.

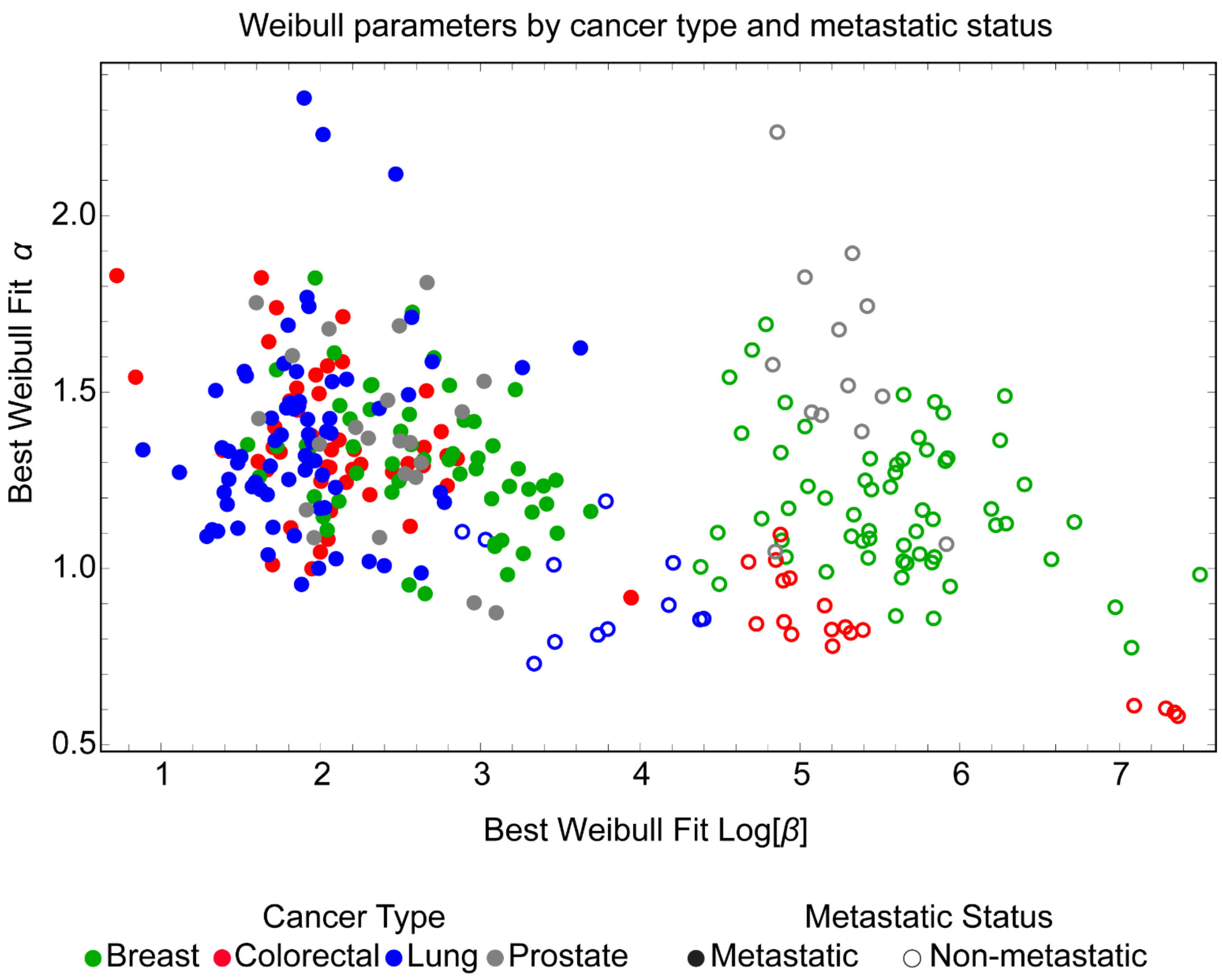


**Supplementary Fig. 8. Best fit Weibull parameter values for trials reporting event-free survival data.** Weibull fits for event-free survival curves labeled by metastatic status and cancer type (encompassing 301 survival curves from 146 trial figures).

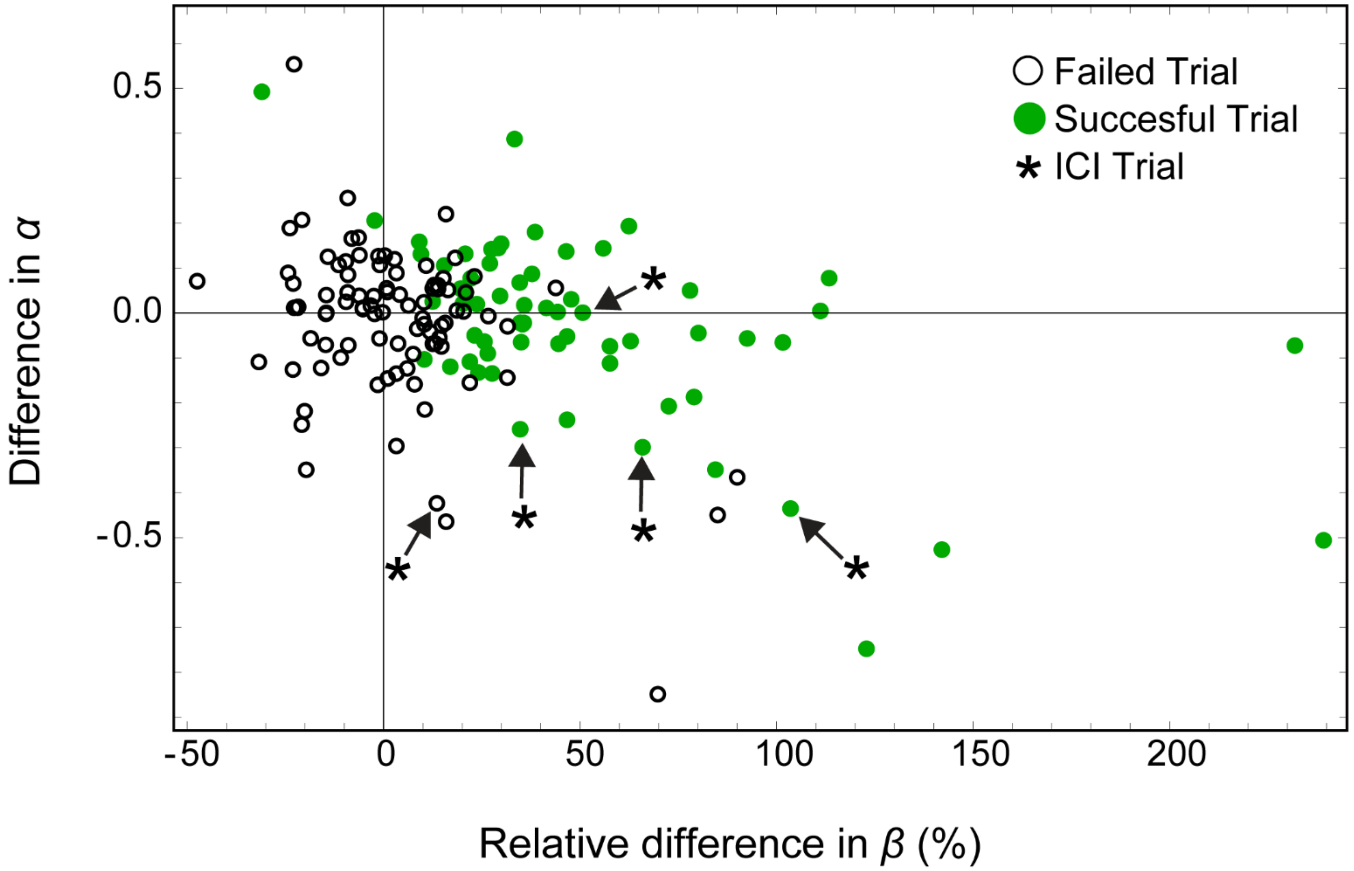


**Supplementary Fig. 9. Parameter values for Weibull fits to event-free survival data scored by trial outcome.** For $\alpha$, the value for the control arm was subtracted from the value for the experimental arm. Differences in $\beta$ were computed by determining the percent change in $\beta$ value in the experimental arm with respect to the control arm (positive values indicate larger $\beta$ in the experimental arm). Success in all cases was judged based on the original report and most often corresponded to a hazard ratio less than one at a 95% confidence level by Cox proportional hazards regression. Asterisks denote trials that tested immune checkpoint inhibitors (ICIs). Data shown for 155 comparisons of experimental and control arms from 146 trial figures.
