# Supplementary material for: Cancer patient survival can be parametrized to improve trial precision and reveal time-dependent therapeutic effects
Source: Nat Commun. 2022 Feb 15;13:873. doi: 10.1038/s41467-022-28410-9 (PMC8847344; doi:10.1038/s41467-022-28410-9)
Supplement: Supplementary file 3 — Description of Additional Supplementary Files [file 41467_2022_28410_MOESM3_ESM.docx]

**DESCRIPTION OF SUPPLEMENTARY DATA**

**Cancer patient survival can be parametrized to improve trial precision and reveal time-dependent therapeutic effects**

Deborah Plana^1,2^, Geoffrey Fell^3^, Brian M. Alexander^3,4^, Adam C. Palmer^5*^, Peter K. Sorger^1*^

^1^Laboratory of Systems Pharmacology and the Department of Systems Biology, Harvard Medical School, Boston, Massachusetts, USA.

^2^Harvard-MIT Division of Health Sciences and Technology, Harvard Medical School and MIT, Cambridge, Massachusetts, USA.

^3^Dana-Farber Cancer Institute, Boston, Massachusetts, USA.

^4^Foundation Medicine Inc., Cambridge, Massachusetts, USA.

^5^Department of Pharmacology, Computational Medicine Program, Lineberger Comprehensive Cancer Center, University of North Carolina at Chapel Hill, Chapel Hill, North Carolina, USA.

^*^These authors contributed equally. To whom correspondence should be addressed:

palmer@unc.edu; peter_sorger@hms.harvard.edu (cc: sorger_admin@hms.harvard.edu)

**Supplementary Data 1.** Clinical trial metadata and individual participant data (IPD). Trial metadata file includes: trial name, author, registration number, journal, publication date, cancer type, cancer metastatic status, whether a significant difference was found between the trial experimental and control arm, treatment name, treatment type, and number of patients enrolled in the trial arm. Comparisons between the imputed trials’ hazard ratios and the original trial hazard ratios are included to assess imputation quality (procedure described in Methods). IPD is provided as 262 .csv files. Each .csv file contains IPD from a different figure from a published clinical trial. Description of all variables included in metadata and .csv files can be found in “README.txt”.

**Supplementary Data 2.** Analysis code and pseudocode. Each piece of code is provided in a folder containing a Mathematica Notebook (.nb), all data required by the code, and the corresponding code output. With source data kept within the same folder as the code, the Mathematica Notebook can be executed in Wolfram Mathematica by selecting “Evaluate Notebook” from the “Evaluation” menu. Pseudocode files summarize the algorithms used to execute analysis corresponding to each result. Sample R code illustrates the parametric fitting and confidence interval construction procedures.

**Supplementary Data 3**. Weibull fitting of immune checkpoint inhibitor (ICI) trial arms. The first tab contains *R^2^* values for one and two-distribution fits of ICI trial arms. The second tab contains the timing of steep drops in survival and the corresponding trial scan times.
