# Supplementary material for: Cancer patient survival can be parametrized to improve trial precision and reveal time-dependent therapeutic effects
Source: Nat Commun. 2022 Feb 15;13:873. doi: 10.1038/s41467-022-28410-9 (PMC8847344; doi:10.1038/s41467-022-28410-9)
Supplement: Supplementary file 5 — Supplementary Dataset 2 [file 41467_2022_28410_MOESM5_ESM.zip › Supplementary Data File 2/Algorithm pseudocode.docx]

**Algorithm 1: Finding best-fit parameter values and computing R^2^ for Weibull fit to cancer survival data.** The same algorithm was executed to obtain the R^2^ for all surrogate even trial figures. Results shown in Figures 1, 2, 5-6, and Supplementary Figures 1-2 and 8-9

**Input:**

*OSTrials=*  descriptor indicating every overall survival (OS) figure for every trial in the data set

*OSTrialsScale =* time scale of reported trial results (i.e.: days, months, weeks, years)

**Execution:**

**For** *i=*1 to Length*[OSTrials]*

*IPD=* extracted individual participant data from .csv file corresponding to OS trial figure

*TherapyNames*= all therapies tested in a given OS trial figure

**For** *j=*1 to Length*[*TherapyNames*]*

*AllTherapyEvents= IPD* events for trial arm in months, normalized using *OSTrialsScale* value

*dist* = Weibull best-fit parameter values for *AllTherapyEvents* calculated using the

“EstimatedDistribution” function with a maximum likelihood approach

*AlphasOS*= Weibull best-fit *α* parameter value for observed data, from *dist*

*BetasOS*= Weibull best-fit *β* parameter value for observed data, from *dist*

*MiddleProgressionTimes*= Moving average of time corresponding to every non-censoring event in a

trial arm (death for OS data)

*SurvivalEstimate*= percent survival at *MiddleProgressionTimes* obtained from traditional

nonparametric survival distribution

// Assessing quality of Weibull fit to survival data using the Weibull Plot

log(-log(Survival)) versus log(time)

*CorrectedS* = Log[-Log[*SurvivalEstimate*]];

*AllTimes*= Log[*MiddleProgressionTimes*]];

*BetaAdjustedLines* = normalizes *AllTimes* (i.e.: *AllTimes* *-*Log*[BetasOS])*

*AlphaAdjustedLines* = normalizes *CorrectedS* (i.e.: *CorrectedS/* *AlphasOS*)

*AllRSquared* = returns the *R^2^* value for every therapy in every trial figure in the data set, calculated

by using the “LinearModelFit” function on *AlphaAdjustedLines*

**Output:**

*OSPlot* = Plot of every *AlphaAdjustedLines* as compared to a linear fit; linear fit represents a Weibull distribution perfectly describing the observed trial data

Median[*AllRSquared*]

Histogram[*AllRSquared*]

**Algorithm 2: Weibull two-parameter fits with cure-rate.** Results shown in Supplementary Fig. 3.

**Input:**

*AllEvents =* all event times and censoring labels corresponding to treatment and trial of interest

**Execution:**

*CensoringEventTimes =* select censoring events

*ProgressionEventTimes =* select deaths or surrogate events

*LogLikelihoodOfCensoringEvents[CensoringEventTimes, alpha, beta]* = for given *α* and *β* values, sum the log-likelihoods for all *n* censoring event times (*t_1_, t_2_, …t_n_*):

$$\sum_{i=1}^{n} -\left( \frac{t_{i}}{\beta} \right)^{\alpha}$$

*LogLikelihoodOfProgressionEvents [ProgressionEventTimes, alpha, beta]* = for given *α* and *β* values, sum the log-likelihoods for all *n* death or surrogate event times (*t_1_, t_2_, …t_n_*):

$$\sum_{i=1}^{n} \left( ln\left( t_{i}^{\alpha-1}\times\alpha\times\beta^{-\alpha} \right)-\left( \frac{t_{i}}{\beta} \right)^{\alpha} \right)$$

*LogLikelihoodOfAllEvents [AllEvents, alpha, beta]=* sums output of *LogLikelihoodOfCensoringEvents* and *LogLikelihoodOfProgressionEvents*

*LogLikelihoodOfCensoringEventsWithCure[CensoringEventTimes, alpha, beta]* = for given values of *α*, *β*, and cure rate ω, sums of the log-likelihoods for all *n* censoring event times (*t_1_, t_2_, …t_n_*) :

$$\sum_{i=1}^{n} ln\left( 1-((1-ⅇ^{-\left( \frac{t_{i}}{\beta} \right)^{\alpha}})\times\left( 1-\omega\right)) \right)$$

*LogLikelihoodOfProgressionEventsWithCure [ProgressionEventTimes, alpha, beta]* = for given values of *α*, *β*, and cure rate ω, sums of the log-likelihoods for all *n* death or surrogate event times (*t_1_, t_2_, …t_n_*):

$$\sum_{i=1}^{n} \left( ln\left( ⅇ^{-\left( \frac{t_{i}}{\beta} \right)^{\alpha}}\times t_{i}^{\alpha-1}\times\alpha\times\beta^{-\alpha}\times(1-\omega) \right) \right)$$

*LogLikelihoodOfAllEventsWithCure [AllEvents, alpha, beta]=* sums output of *LogLikelihoodOfCensoringEventsWithCure* and *LogLikelihoodOfProgressionEventsWithCure*

*TableOfLogLikelihoods =* calculates *LogLikelihoodOfAllEvents* across a 2D matrix of *α* and *β* parameter values

*TableOfLogLikelihoodsWithCure =* calculates *LogLikelihoodOfAllEventsWithCure* across a 3D matrix of *α*, *β,* and cure rate ω parameter values

**Output:**

*BestFit2Param*= set of Weibull parameters with highest likelihood from *TableOfLogLikelihoods*

*BestFitParameters*= set of Weibull parameters with highest likelihood from *TableOfLogLikelihoodsWithCure*

*DataAndFitsOverlay*= plot of trial data, Weibull two-parameter fit using *BestFit2Param* values*, and* two-parameter fit with cure-rate ω using values using *BestFitParameters* values:

$$\text{Survival}\left( t \right)=\omega+\left( 1-\omega\right)\times\left( ⅇ^{-\left( \frac{t}{\beta} \right)^{\alpha}} \right)$$

**Algorithm 3A: Fitting of Weibull and Weibull mixture models to survival for immune-checkpoint trials.** Results shown in Figure 3 and Supplementary Fig. 4.

**Input:**

*TherapyEvents=* all event times and censoring labels corresponding to treatment and trial of interest

**Execution**:

*W1=* set of Weibull parameters estimated using the “EstimatedDistribution” function with a maximum likelihood approach

*W2*= set of Weibull mixture model parameters estimated using the “EstimatedDistribution” function with a maximum likelihood approach

*AverageProgression*= moving average of times corresponding to every death or progression in trial arm

*ObservedSurvival*= observed survival at each *AverageProgression* time

*W1Survival*= estimated survival at each *AverageProgression* time from Weibull distribution with *W1* parameters

*W2Survival*= estimated survival at each *AverageProgression* time from Weibull mixture model with *W2* parameters

**Output:**

*ICI=* plot showing original trial data along with Weibull distribution and Weibull mixture model fits (using *W1* and *W2* parameter values)

*R^2^* value for relationship between *W1Survival* and *ObservedSurvival* obtained using the “LinearModelFit” function

*R^2^* value for relationship between *W2Survival* and *ObservedSurvival* obtained using the “LinearModelFit” function

**Input:**

*Events=* all event times and censoring labels corresponding to treatment and trial of interest

*ScanInterval=* scan time periodicity in months, obtained from published trial protocol

*ScanStDev*= standard deviation of scan time in population of patients, estimated from visual inspection of trial data

**Execution**:

*DistributionFit=* set of Weibull parameters estimated using the “EstimatedDistribution” function with a maximum likelihood approach

*PatientsScanTimes=* samples from normal distribution of possible scan times, with a mean equal to the expected scan time from the trial protocol and a standard deviation of *ScanStDev*

*PatientsProgressionTimes* = expected progression events generated by sampling from Weibull distribution

*PatientProgressionObservationTimes=* the expected scan times; calculated by finding the value from *PatientsScanTimes* that is most recently after each *PatientsProgressionTimes* event

*PFSfunction* = generates empirical survival function from *PatientProgressionObservationTimes*

*AverageProgression*= moving average of times corresponding to every progression in trial arm

*ObservedSurvival*= observed survival at each *AverageProgression* time

*ScanSurvival*= estimated survival at each *AverageProgression* time from *PFSfunction*

*WSurvival*= estimated survival at each *AverageProgression* time from Weibull fit using *DistributionFit* parameter values

**Output:**

*MyPlot =* plot showing original trial data along with fits from *PFSfunction* and *DistributionFit*

*R^2^* value for relationship between *WSurvival* and *ObservedSurvival* obtained from the “LinearModelFit” function

*R^2^* value for relationship between *ScanSurvival* and *ObservedSurvival* obtained from the “LinearModelFit” function

**Algorithm 3B: Simulating the periodicity of radiological scans to fit survival from immune-checkpoint inhibitor trials.** Results shown in Figure 3 and Supplementary Fig. 4.

**Algorithm 4: Computing parametric and nonparametric confidence intervals for subsampled Phase 3 trials.** Results shown in Figure 4 and Supplementary Fig. 5-6.

**Input:**

*Cutoffs* = patient cohort subsampling sizes, ranging from 20 to 100 with a step size of 10

*TimeExp* = time point of interest for confidence interval generation (12 months)

**Execution**:

*UniqueFig*= metadata corresponding to each trial figure

**For** *i=*1 to Length*[UniqueFig]*

*ArmstoCompare*= metadata corresponding to each arm of each trial figure

**For** *m =* 1 to Length*[ArmstoCompare]*

**If** the number of patients in a trial arm is at least 100 and there was at least one trial arm event at or after

*TimeExp*

**For** *k=*1 to Length*[Cutoffs]*

*SmallSample* = randomly sample number of patient events in *Cutoffs* from trial arm data

**While** the total number of non-censoring events is less than 3, draw another *SmallSample*

//Parametric confidence interval calculation

*T1deathevents =* progression or surrogate events from *SmallSample*

*T1censorevents=* censoring events from *SmallSample*

*T1LogSumOfProbabilities=* for given *α* and *β* values, sum of the log-likelihoods for all death or surrogate events (computed as the PDF at each event time) and of all censoring events (computed as 1- CDF at each censoring time)

*TableOfLogLikelihoods =* calculates *T1LogSumOfProbabilities* across a set of *α* (0.1 to 4) and *β* values (1 to 2*the best fit Weibull *β* value) for *SmallSample* event data

*T1maxP*= maximum likelihood from *TableOfLogLikelihoods*

*T1LikelihoodWeightedParameters*= sorts *TableOfLogLikelihoods* by likelihood

(normalized by e^(*- T1maxP* +log-likelihood)

*SampleSize*=100

*T1LikelihoodWeightedParametersSelected*= selects for values of *T1LikelihoodWeightedParameters*

with likelihood greater or equal to 1/ *SampleSize*

*T1ParameterSamples=* samples parameters from *T1LikelihoodWeightedParametersSelected* proportionally to their likelihood

*WeibullSim*= calculates expected survival at *TimeExp* from 1- CDF of the Weibull distribution with parameters from *T1ParameterSamples*

**Algorithm 4 continued: Computing parametric and nonparametric confidence intervals for subsampled Phase 3 trials.** Results shown in Figure 4 and Supplementary Fig. 5-6.

**Output:**

*YearSurvivalCurrent* = percent survival at *TimeExp* for full Phase 3 trial data (ground truth)

*CINPs*= nonparametric confidence intervals for *SmallSample* at *TimeExp*, calculated using the "PointwiseBands" property from the “SurvivalModelFit” function

*YearSurvivalNPs* = nonparametric survival estimate for *SmallSample* at *TimeExp*

*Wbound*= upper and lower 95% Weibull confidence intervals for survival at *TimeExp* for each *SmallSample*, calculated by taking the 2.5% and 97.5% quantiles value from *WeibullSim* using the “Quantile” function

*Sig50*= Weibull estimate of survival at *TimeExp* for each *SmallSample*, calculated as the mean of *WeibullSim*

**Input:**

*Trialdata*= extracted individual participant data from .csv file corresponding to OS trial figure

**Execution:**

*SampleSize*=10,000

…

*// T1ParameterSamples* and *T2ParameterSamples* were computed using Phase 3 trial arm data per the procedure described in Algorithm 4

*T1ParameterSamples*= likelihood-weighted sample of *α* and *β* parameter values for the control treatment arm

*T2ParameterSamples*= likelihood-weighted sample of *α* and *β* parameter values for the experimental treatment arm

*CumulativeHazardRatioSamples* = takes the ratio of the Weibull Cumulative Hazard$(({\frac{t}{\beta})}^{\alpha})$ evaluated for parameters in *T1ParameterSamples* over the Weibull Cumulative Hazard evaluated for parameters in *T2ParameterSamples*

*CumulativeHazardRatioConfidenceIntervals*= computes the mean as well as upper and lower 95% confidence interval values for *CumulativeHazardRatioSamples* over a range of trial times

**Output:**

Plot of the mean, upper, and lower confidence interval values from *CumulativeHazardRatioConfidenceIntervals*. Compares to the reported Cox regression hazard ratio and associated 95% confidence interval.

**Algorithm 5: Comparing the Weibull ratio of cumulative hazards and the Cox regression hazard ratio.** Results shown in Figure 6.

**Algorithm 6: Simulating the effect of trial duration on success.** Results shown in Figure 7.

**Input:**

*Alphas=* range of experimental arm *α* values for simulation (0.5-4.5 with a step size of 0.25)

*Betas=* range of experimental arm *β* values for simulation (0.4-1.6 with a step size of 0.1)

*Alpha1=* control arm *α* value (1.5)

*Beta1=* control arm *β* value (1)

*Iterations* = 1000

*CensoringDuration* = last follow-up time in simulated trial. Two sets of simulations were run for follow-up times of *Beta1* and 4**Beta1* respectively

**Execution**:

**For** *i*=1 to *Iterations*,

*PatientEventsControl*= randomly sample 100 patient events for the simulated control arm

using a Weibull distribution with parameters *Alpha1* and *Beta1.* Censor any event that

takes place after *CensoringDuration*

**For** *l*=1 to Length*[Alphas]*,

*Alpha2* = *Alphas*[[*l*]]

**For** *j*=1 to Length*[Betas]*,

*Beta2* = *Betas*[[*j*]]

*PatientEventsExp*= randomly sample 100 patient events for the simulated

experimental arm using a Weibull distribution with parameters *Alpha2* and *Beta2.*

Censor any event that takes place after *CensoringDuration.*

**Output:**

*RR*= relative risk returned from comparing the *PatientEventsControl* and

*PatientEventsExp* simulated trial arms using the “CoxModelFit” function

*PV*= *p* value associated with “CoxModelFit” output, calculated using a Wald test

**Algorithm 7: Simulating and assessing performance of Weibull fitting on trial arms subsampled from heterogeneous cancer trials.** Results shown in Supplementary Fig. 7.

**Input:**

*OSTrials=*  descriptor indicating every overall survival (OS) figure for every trial in the data set

*OSTrialsScale =* time scale of reported trial results (i.e.: days, months, weeks, years)

**Execution:**

**For** *i=*1 to Length*[OSTrials]*

*TherapyNames*= all therapies tested in a given OS trial figure

// For metastatic cancer simulation, only proceeds with execution if trial metadata indicates

that the trial included metastatic cancer patients

**For** *j=*1 to Length*[*TherapyNames*]*

*IPDTherapy=* events for trial arm in months, normalized using *OSTrialsScale* value

*SimPatients*= randomly sample five patient events from *IPDTherapy*

*AllPatientsSim*= list of *SimPatients* for every trial arm; all patient events for simulated cohort

// Parameter estimation and *R^2^* value calculation for Weibull two-parameter fit on event data from *AllPatientsSim* performed as described in Algorithm 1A

// Parameter estimation for Weibull cure-rate model on event data from *AllPatientsSim* was performed as described in Algorithm 2

// *R^2^* calculation for cure-rate parameter fit described below

*MiddleProgressionTimes*= Moving average of time corresponding to every non-censoring event in *AllPatientsSim*

*SurvivalEstimate*= percent survival at *MiddleProgressionTimes* obtained from traditional

nonparametric survival distribution, then scaled using the cure-rate parameter ((survival probability- cure rate)/(1-cure rate))

*CorrectedS* = Log[-Log[*SurvivalEstimate*]];

*AllTimes*= Log[*MiddleProgressionTimes*]];

*BetaAdjustedLines* = normalizes *AllTimes* (i.e.: *AllTimes* *-*Log*[BetasOS])*

*AlphaAdjustedLines* = normalizes *CorrectedS* (i.e.: *CorrectedS/* *AlphasOS*)

*AllRSquared* = returns the *R^2^* value for the Weibull cure-rate model by using the “LinearModelFit” function on *AlphaAdjustedLines*

**Output:**

Plot of simulated patient cohort, Weibull two-parameter fit, and Weibull two-parameter fit with cure-rate
