# Supplementary figures and images for: Cancer patient survival can be parametrized to improve trial precision and reveal time-dependent therapeutic effects
Source: Nat Commun. 2022 Feb 15;13:873. doi: 10.1038/s41467-022-28410-9 (PMC8847344; doi:10.1038/s41467-022-28410-9)

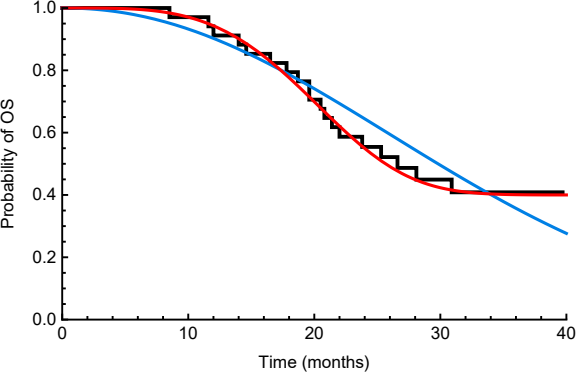

Supplement: Supplementary file 5 — Supplementary Dataset 2 [file 41467_2022_28410_MOESM5_ESM.zip › Supplementary Data File 2/3 parameter fits- Fig S3/ACT_2D/ACT2_2D.pdf]

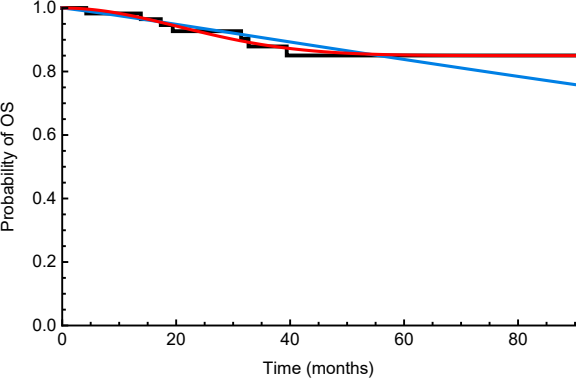

Supplement: Supplementary file 5 — Supplementary Dataset 2 [file 41467_2022_28410_MOESM5_ESM.zip › Supplementary Data File 2/3 parameter fits- Fig S3/Chronicle_2B/Chronicle_2B.pdf]
