# Supplementary material for: Cancer patient survival can be parametrized to improve trial precision and reveal time-dependent therapeutic effects
Source: Nat Commun. 2022 Feb 15;13:873. doi: 10.1038/s41467-022-28410-9 (PMC8847344; doi:10.1038/s41467-022-28410-9)
Supplement: Supplementary file 7 — Reporting Summary [file 41467_2022_28410_MOESM7_ESM.pdf]

nature portfolio

Corresponding author(s): Adam C. Palmer and Peter K. Sorger

Last updated by author(s): Dec 14, 2021

Reporting Summary

Nature Portfolio wishes to improve the reproducibility of the work that we publish. This form provides structure for consistency and transparency in reporting. For further information on Nature Portfolio policies, see our Editorial Policies and the Editorial Policy Checklist.

Statistics

For all statistical analyses, confirm that the following items are present in the figure legend, table legend, main text, or Methods section.

n/a	Confirmed	
☐	☒	The exact sample size (*n*) for each experimental group/condition, given as a discrete number and unit of measurement
☒	☐	A statement on whether measurements were taken from distinct samples or whether the same sample was measured repeatedly
☐	☒	The statistical test(s) used AND whether they are one- or two-sided<br>*Only common tests should be described solely by name; describe more complex techniques in the Methods section.*
☒	☐	A description of all covariates tested
☐	☒	A description of any assumptions or corrections, such as tests of normality and adjustment for multiple comparisons
☐	☒	A full description of the statistical parameters including central tendency (e.g. means) or other basic estimates (e.g. regression coefficient) AND variation (e.g. standard deviation) or associated estimates of uncertainty (e.g. confidence intervals)
☐	☒	For null hypothesis testing, the test statistic (e.g. *F*, *t*, *r*) with confidence intervals, effect sizes, degrees of freedom and *P* value noted<br>*Give P values as exact values whenever suitable.*
☒	☐	For Bayesian analysis, information on the choice of priors and Markov chain Monte Carlo settings
☒	☐	For hierarchical and complex designs, identification of the appropriate level for tests and full reporting of outcomes
☐	☒	Estimates of effect sizes (e.g. Cohen's *d*, Pearson's *r*), indicating how they were calculated

Our web collection on statistics for biologists contains articles on many of the points above.

Software and code

Policy information about availability of computer code

Data collection	Plots of the Kaplan Meier estimator were extracted from trial figures using the DigitizeIt software (version 2.5.3).
Data analysis	Analysis was performed using Wolfram Mathematica Version 12.1. Compute-intensive analyses (e.g. sample size simulations) were conducted on the O2 High Performance Compute Cluster, supported by the Research Computing Group, at Harvard Medical School.<br>All code used in this study is included in Supplementary Data 2. Code is also available through Synapse (ID: syn25813713). Each piece of code is provided in a folder containing a Mathematica Notebook (.nb), all data required by the code, and the corresponding code output. With source data kept within the same folder as the code, the Mathematica Notebook can be executed in Wolfram Mathematica by selecting “Evaluate Notebook” from the “Evaluation” menu.<br>Sample R code (R version 4.0.3) illustrates the parametric fitting and confidence interval construction procedures.<br>Pseudocode files summarize the algorithms used to execute analysis corresponding to each result.

For manuscripts utilizing custom algorithms or software that are central to the research but not yet described in published literature, software must be made available to editors and reviewers. We strongly encourage code deposition in a community repository (e.g. GitHub). See the Nature Portfolio guidelines for submitting code & software for further information.

Data

Policy information about availability of data

All manuscripts must include a data availability statement. This statement should provide the following information, where applicable:

- Accession codes, unique identifiers, or web links for publicly available datasets
- A description of any restrictions on data availability
- For clinical datasets or third party data, please ensure that the statement adheres to our policy

All data generated or analyzed during this study are included in this published article (and its supplementary information files). Data are also available through the website https://cancertrials.io./ and Synapse (ID: syn25813713).

Field-specific reporting

Please select the one below that is the best fit for your research. If you are not sure, read the appropriate sections before making your selection.

☒ Life sciences ☐ Behavioural & social sciences ☐ Ecological, evolutionary & environmental sciences

For a reference copy of the document with all sections, see nature.com/documents/nr-reporting-summary-flat.pdf

Life sciences study design

All studies must disclose on these points even when the disclosure is negative.

Sample size	The original data set consisted of 153 unique trials in breast, colorectal, lung, and prostate cancer in the metastatic and non-metastatic settings from 2014-2016 that met desired search criteria. For additional information on study selection, data extraction, and reconstruction procedures, see: Fell, G., Redd, R.A., Vanderbeek, A.M., Rahman, R., Louv, B., McDunn, J., Arfè, A., Alexander,B.M., Ventz, S., Trippa, L. (2021). KMDATA: a curated database of reconstructed individual patient-level data from 153 oncology clinical trials, Database, Volume 2021, 2021, baab037.<br><br>No new clinical trials were performed as part of the study. Sample size information for specific studies is described in the original trial publications (list in Supplementary Data 1).
Data exclusions	Trials were removed from the original data set if there were any inconsistencies in the imputed patient data as compared to its associated clinical trial (e.g.: differing numbers of patients from the publication at-risk table and imputed data). The quality of the data imputation was confirmed quantitatively, by calculating the hazard ratio for imputed data and comparing it to the corresponding trial's reported hazard ratio, and qualitatively, by overlaying the Kaplan-Meier curve generated from the imputed data on top of the published curve. Trials with a hazard ratio difference greater than 0.1, or with perceptible visual differences, were removed from the final data set and not analyzed further.
Replication	All code was re-executed in preparation for manuscript submission and the reproducibility of the results were confirmed. Each piece of code is provided in a folder containing a Mathematica Notebook (.nb), all data required by the code, and the corresponding code output (Supplementary Data 2). With source data kept within the same folder as the code, the Mathematica Notebook can be executed in Wolfram Mathematica by selecting "Evaluate Notebook" from the "Evaluation" menu.
Randomization	Randomization was not relevant to our study as the work consisted of a re-analysis of existing clinical trial data. No new clinical trials were performed as part of the study. Randomization information for specific studies is described in the original trial publications (list in Supplementary Data 1).
Blinding	Blinding was not relevant to our study as the work consisted of a re-analysis of existing clinical trial data. No new clinical trials were performed as part of the study. Blinding information for specific studies is described in the original trial publications (list in Supplementary Data 1).

Reporting for specific materials, systems and methods

We require information from authors about some types of materials, experimental systems and methods used in many studies. Here, indicate whether each material, system or method listed is relevant to your study. If you are not sure if a list item applies to your research, read the appropriate section before selecting a response.

Materials & experimental systems

n/a	Involved in the study	
☒	☐	Antibodies
☒	☐	Eukaryotic cell lines
☒	☐	Palaeontology and archaeology
☒	☐	Animals and other organisms
☐	☒	Human research participants
☐	☒	Clinical data
☒	☐	Dual use research of concern

Methods

n/a	Involved in the study	
☒	☐	ChIP-seq
☒	☐	Flow cytometry
☒	☐	MRI-based neuroimaging

Human research participants

Policy information about studies involving human research participants

Population characteristics	Imputation of individual participant data only generates de-identified (anonymous) survival data from published sources; this is not Human Subjects Research per Code of Federal Regulations 45 CFR 46 Subpart A.<br><br>Population characteristics are described in the original trial publications (list in Supplementary Data 1).
Recruitment	Recruitment methods are described in the original trial publications (list in Supplementary Data 1).
Ethics oversight	All trial analyzed in this manuscript have been previously published (list in Supplementary Data 1). Any information that could lead to the identification of an individual patient was not accessed, and no concerning ethical issue was raised in this research that would necessitate ethical approval or participant consent. Ethics oversight information for individual trials can be found in the original trial publications.

Note that full information on the approval of the study protocol must also be provided in the manuscript.

Clinical data

Policy information about clinical studies

All manuscripts should comply with the ICMJEguidelines for publication of clinical research and a completedCONSORT checklist must be included with all submissions.

Clinical trial registration	We did not conduct the clinical trials analyzed in the article, had no role in the approval of the trial protocols, and have no further information on their clinical data than any member of the public who reads the articles.<br><br>Clinical trial registration information is described in the original trial publications (list in Supplementary Data 1).
Study protocol	Study protocol information is described in the original trial publications (list in Supplementary Data 1).
Data collection	Data collection procedures are described in the original trial publications (list in Supplementary Data 1).
Outcomes	Outcome measures are described in the original trial publications (list in Supplementary Data 1).
